# Not all developmental assets are related to positive health outcomes in college students

**DOI:** 10.1186/1477-7525-9-52

**Published:** 2011-07-13

**Authors:** Keith J Zullig, Daniel A Teoli, Rose Marie Ward

**Affiliations:** 1 Department of Community Medicine, West Virginia University, Morgantown, WV, USA; 2 Department of Kinesiology and Health, Miami University of Ohio, Oxford, OH, USA

## Abstract

**Background:**

The purpose of this investigation was to model the relationships between developmental assets, life satisfaction, and health-related quality of life (HRQOL) among a stratified, random sample (*n *= 765, 56% response rate) of college students.

**Methods:**

Structural equation modeling techniques were employed to test the relationships using Mplus v4.21; Model evaluations were based on 1) theoretical salience, 2) global fit indices (chi-square goodness of fit, comparative fit index: CFI and Tucker-Lewis Index: TLI), 3) microfit indices (parameter estimates, root mean squared error of approximation: RMSEA and residuals) and 4) parsimony.

**Results:**

The model fit the data well: χ^2^(*n *= 581, 515) = 1252.23, CFI = .94, TLI = .93 and RMSEA = .05. First, participants who reported increased Family Communication also reported higher levels of life satisfaction. Second, as participants reported having more Non-Parental Role Models, life satisfaction decreased and poor mental HRQOL days increased. Finally increased Future Aspirations was related to increased poor mental HRQOL days. Results were variant across gender.

**Conclusions:**

Preliminary results suggest not all developmental assets are related to positive health outcomes among college students, particularly mental health outcomes. While the findings for Family Communication were expected, the findings for Non-Parental Role Models suggest interactions with potential role models in college settings may be naturally less supportive. Future Aspirations findings suggest college students may harbor a greater temporal urgency for the rigors of an increasingly competitive work world. In both cases, these assets appear associated with increased poor mental HRQOL days.

## Background

Positive youth development (PYD) first originated as a conceptual approach of developing assets within youth as opposed to removing risk factors via "deficit-focused strategies" [1]. Evidence suggests a relationship exists between the number of assets possessed and the number of thriving indicators within an individual (such as possession of leadership qualities, display of resiliency, and achieved success in school) [2]. Building upon this support, PYD considers the strengths of youth and values the contributions they can make toward healthy development by maximizing these individual strengths through meaningful societal roles and community-based activities [3].

PYD is often assessed through the Search Institute's Developmental Asset Framework [4]. This framework suggests that 40 primary assets may affect healthy youth development. These assets are grouped into either internal or external assets. Internal assets are skills, values, and commitments that stem from within an individual including (but not limited to) humility, appropriate decision-making, and a sense for his or her own purpose in life. Internal assets categories include commitment to learning, positive values, social competencies, or positive identity. In contrast, external assets develop outside of an individual. External assets are positive experiences and interactions gained from one's family, non-parental role models, school, community, and service groups. External asset categories include support, empowerment, boundaries and expectations, or constructive use of time [5]. When the internal and external assets are in a balanced positive state of existence, PYD can occur [6].

Increased asset development may serve as an important protective factor for individuals [7]. For example, those who possess increased developmental assets are less likely to report violent and aggressive behavior [8]; tobacco use [9]; risky sexual behavior [10,11]; and alcohol and drug use [12]. These findings may be especially pertinent to college students, who constitute a population often exposed to unusual stressors such as living in a new location, pressure for high academic achievement, immediate availability of illegal substances, and increased risk of dangerous sexual behaviors. These stressors can effectively serve as barriers to a smooth transition for students from their homes to an independent college environment [13,14]. Moreover, research also suggests that the total number of assets possessed by a student is approximately two times more effective in anticipating future achievement than are other predictive measures (e.g., race/ethnicity, family composition and socioeconomic status) [3]. For instance, the correlations between academic achievement (GPA) and the total number of assets in an individual was .45 for males and .35 for females in a recent study [15].

While developmental assets appear to be protective against engagement in risky behaviors, little is known about the relationship between developmental assets and one's quality of life (QOL). According to Diener [16], improving QOL is important for enriching an individual's overall well-being. QOL consists of two dimensions: subjective and objective. Objective QOL examines issues external to an individual such as annual income level, neighborhood crime rates, and personal housing quality. Alternatively, subjective QOL consists of judgments of one's overall life in different domains including, but not limited to, self, family life, and romantic relationships. Subjective QOL can be further separated into life satisfaction and health-related quality of life (HRQOL) [17]. Specifically, life satisfaction is a cognitive conclusion regarding the quality of one's own life in comparison with a self-imposed standard [18]. The outcome of comparing actual circumstances vis-à-vis personal standards will render individual global life satisfaction judgments as positive or negative in nature [19]. The United States (US) Centers for Disease Control and Prevention (CDC) define HRQOL as "an individual's or group's perceived physical and mental health over time" [20]. On the micro scale, HRQOL observes physical and mental health levels of an individual. On the macro scale, an entire population (or student body) can be analyzed for the impact that policies, resources, and conditions (i.e., developmental assets) have upon that respective population's health.

Similar to a lack of developmental assets [8,9,11,12], lower levels of life satisfaction are related to increased violent and aggressive behavior [21], substance use [22], and risky sexual behavior [23]. Reduced life satisfaction is also related to unhealthy dieting and weight perceptions [24-26], suicide ideation [27] and a sedentary lifestyle [28]. In light of these established relationships with life satisfaction, subjective quality of life is an important facet of health research that is often overlooked [29]. Unfortunately, the literature contains little research exploring the relationship between life satisfaction and developmental assets in the context of PYD. While one study by Valois et al. [30] indicates a significant positive relationship between increased developmental assets and life satisfaction, the findings are limited to one study of public middle school students. No studies examine a college student population. Therefore, one aim of this study is to explore the relationship between developmental assets and college student life satisfaction.

A second aim of this study is to explore the relationship between developmental assets and HRQOL. According to the US CDC [31], "fair or poor" self-rated health was reported by 9.7% of 18-24 year olds in 2007 - an increase from 6.5% in 1993. Similarly, the "mean physically unhealthy days" (out of the prior 30) was an average of 4.3 days in 2006 and the mean "mentally unhealthy days" (out of the prior 30) was approximately 6.0 days in 2006 [31], both of which have increased since 1996. However, no adult or adolescent literature explores the relationship between developmental assets and HRQOL.

Therefore, the purpose of this study is to explore the concurrent relationships between the developmental assets, life satisfaction, and HRQOL among college students. The need for a more thorough understanding of these relationships is further bolstered by two important findings: the current generation of teenagers exhibit overall worse mental health [32] and higher levels of anxiety [33] in comparison to previous generations. The current study extends the extant literature by 1) exploring any relationships between the developmental assets, life satisfaction, and HRQOL outcomes and by 2) suggesting which developmental assets are most strongly related to both life satisfaction and HRQOL. This research may be subsequently used to direct PYD approaches in college settings.

## Methods

### Sampling Method

During February 2007, 1,300 students 18 years of age or older were randomly selected from a Midwestern university's email database to participate in an internet-based health survey. Equal numbers of students from each academic class were selected at random (i.e. a stratified random sample) via the uniform distribution number generator function in SAS [34]. The methodology provided each student an equal probability of being selected as a participant in the investigation's sample to produce a representative sample of the university's students. The sample database included student names, up-to-date mailing addresses, and current email addresses.

Using internet survey methods [35] approved by the referent university's institutional review board, selected students (*n *= 1,300) were sent an invitation to participate. Selected students were notified that if they participated in the investigation, they would receive a coupon to an off-campus café (redeemable for a single specialty drink of choice). Seven days after the initial solicitation, a second email containing a clickable hyperlink to the questionnaire was sent to the potential participants. Those who clicked the hyperlink were first directed to an informed consent statement that explained the pertinent research procedures and specific active measures being taken to protect participants' privacy. At the conclusion of the survey, all potentially identifying information details were separated from the responses and stored in a separate data storage location (thus making responses anonymous). A total of 723 surveys were completed for a 56% response rate.

### Participants

Sample demographics are provided in Table 1. The referent institution is a four-year, public university of midsize in the Midwestern United States. Approximately 14,265 students of the 16,262 total student population are Caucasian (86%) and only 2% of students are above the age of 24. The composition of the University's undergraduate body is 46% male and 54% female. The percentages of freshman, sophomore, junior and senior students comprising the student body are 27%, 27%, 21% and 24%, respectively. While females are slightly overrepresented and males are underrepresented in this sample, the racial and academic year demographics are quite representative of the campus as a whole.

**Table 1:** Sample Demographics

Characteristic	**Number of Respondents **^**(N = 723)**^
**Age group (years)**	
18 yrs	93 (12.9%)
19 yrs	172 (23.8%)
20 yrs	177 (24.5%)
21 yrs +	281 (38.9%)
**Year**	
First year	193 (27.7%)
Sophomore	155 (21.4%)
Junior	203 (28.1%)
Senior	172 (23.8%)
**Gender**	
Male	232 (32.1%)
Female	491 (67.9%)
**Race**	
White	654 (90.5%)
Nonwhite	69 (9.5%)

### Measures

#### Developmental Assets

Based on the work of Oman et al. [36], the developmental asset measure used in this study was developed and validated for college students [37]. The survey contains 28 items and measures eight developmental assets. The first asset is "Family Communication" with three items (response options 1 = almost never; 2 = some of the time; 3 = usually; 4 = almost always). A sample Family Communication item is "*How often does your mother, father, or another adult at your home try to understand your point of view?*" The second asset is "Peer Role Models" also with three items (response options 1 = almost never; 2 = some of the time; 3 = usually; 4 = almost always); a sample item is "*Are most of your friends responsible?*" The third asset is "Future Aspirations" with two items (response options 1 = not important at all; 2 = somewhat important; 3 = very important; 4 = extremely important); a sample item is "*As you look into the future, how important is it that you do well in school?*" The fourth asset is "Responsible Choices" with three items (response options 1 = not at all like you; 2 = a little like you; 3 = mostly like you; 4 = very much like you); a sample item is "*You can say 'no' to activities you think are wrong*." The fifth asset is "Non-Parental Adult Role Models" with four items (response options 1 = strongly disagree; 2 = disagree; 3 = agree; 4 = strongly agree); a sample item is "Y*ou know at least one adult on campus you could talk with about personal problems*." The sixth asset is "Spirituality" and contains six items (response options 1 = strongly agree; 2 = agree; 3 = disagree; 4 = strongly disagree); a sample is "*Spirituality is very important to me*." The seventh asset is "Community Involvement" contains four items (response options 1 = not at all like you; 2 = a little like you; 3 = mostly like you; 4 = very much like you); a sample item is "*You work to make your community a better place*." The final asset is "Cultural Respect/Life" contains three items (response options 1 = not at all like you; 2 = a little like you; 3 = mostly like you; 4 = very much like you); a sample item is "*You respect the beliefs of people even if they are of a different race*." In this study, the Cronbach's alphas for the eight asset subscales are .76 (Family Communication), .80 (Peer Role Models), .55 (Future Aspirations), .71 (Responsible Choices), .74 (Non-Parental Role Models), .90 (Spirituality), .88 (Community Involvement), and .76 (Cultural Respect/Life).

#### Brief Multidimensional Students' Life Satisfaction Scale (BMSLSS-C)

The BMSLSS-C consists of one item for each of 7 life satisfaction domains (i.e., family, friends, school, self, living environment, romantic relationships, physical appearance) determined to be valid and reliable in college students [38]. The item assessing satisfaction in one's family life is "*I would describe my satisfaction with my family life as*," whereas "*I would describe my satisfaction with my physical appearance *as" assesses physical appearance satisfaction and so forth. Response options are from the widely used Delighted-Terrible Scale [39] and include 1 = terrible, 2 = unhappy, 3 = mostly dissatisfied, 4 = mixed (about equally satisfied and dissatisfied), 5 = mostly satisfied, 6 = pleased, and 7 = delighted. Although a global life satisfaction item can also be used as a part of the BMSLSS-C, it was not included in the study due to redundancy. The Cronbach's alpha for BMSLSS-C in this study is .80.

#### The Centers for Disease Control and Prevention's HRQOL-14

The HRQOL-14 is based on research with adults age 18 or older. The original survey consisted of four core questions on the Behavioral Risk Factor Surveillance System (BRFSS) [40,41]. Item 1 focuses on self-perceived health with response options of "excellent," "very good," "good," "fair," and "poor." Items 2 and 3 relate to recent physical and mental health symptoms and are considered mutually exclusive and were worded as such: "*Now thinking about your physical (or mental) health, for how many days during the past 30 days was your physical (or mental) health not good?*" Item 4 is conceptualized as a global measure of disability that explicitly incorporates both physical and mental health: "*During the past 30 days, on how many days did poor physical or mental health keep you from doing your usual activities ...?*" In 1995, an optional 10-item set of health perception and activity limitation items was added to the BRFSS. These items cover areas such as sleep, anxiousness/worrying, pain, and feeling full of energy (all during the past 30 days). All response options to the scale "days" items were identical and assessed the number of days that symptoms were experienced: 0 days, 1-2 days, 3 to 5 days, 6 to 9 days, 10 to 19 days, 20 to 29 days, and all 30 days.

Hennessey et al. [41] originally established the construct validity of the four core questions. Additional validation research has revealed that the HRQOL-14 demonstrated good construct, criterion, and known-groups validity and that it could be considered for both surveillance and research applications when compared to the Rand Corporation's Short Form-36 (SF-36) [42]. The SF-36[43] is generally considered the "gold standard" for quality of life (QOL) research. Other validity research found the HRQOL-14 identified known or suspected population groups with unmet health-related needs, including those who reported chronic health conditions, disabilities, and low socioeconomic status (SES) [44-46]. The HRQOL scale also exhibits validity among college students [47]. Reliability studies reveal considerable test-retest reliability [48,49].

### Data Analysis

#### Analysis Plan

Multiple structural equation models (SEM) examined the proposed relationships among the developmental assets, life satisfaction, and HRQOL. The relationships between the constructs were assessed using Mplus version 4.21 [50] using maximum likelihood estimation. SEM procedures were selected for this investigation because they offer several advantages over traditional multivariate methods (e.g., ANOVA, MANOVA, etc.). First, because most outcomes (HRQOL and life satisfaction) have multiple predictors (developmental assets) that interact, SEM examines dependent and independent variables at once. Second, SEM procedures allow for the inspection of relationships among latent variables (underlying, but not directly measured) and multiple observed measures.

Models were proposed based upon theoretical predictions and examined using the following criteria: (1) theoretical salience, (2) global fit indices (chi-square goodness of fit, Comparative Fit Index: CFI & Tucker-Lewis Index: TLI), (3) microfit indices (parameter estimates, Root Mean Squared Error of Approximation: RMSEA, and residuals), and (4) parsimony. Theoretical fit was examined with respect to documented theory and previous research. For the global fit indices, a non-significant chi-square indicates that the data does not significantly differ from the hypotheses represented by the model; for CFI and TLI, fit indices of above 0.90 (preferably above 0.95) are the criteria utilized to indicate a well-fitting model (CFI: [51]; TLI: [51]). For RMSEA, a fit of less than 0.05 indicates a well-fitting model [52]. Finally, requiring parsimony leads to the retention of a model with the fewest parameters that still meet the other criteria.

## Results

### Descriptive Statistics

#### HRQOL

A majority of the participants reported "excellent" or "very good" self-rated health (70.28%). Days in the past month where the participants' mental health was not good were: 0 days - 21.71%; 1-2 days - 32.03%; 3-5 days - 21.31%; 6+ days 22.95%. Days in the past month where the participants' physical health was not good were: 0 days - 24.21%; 1-2 days - 36.32%; 3-5 days - 22.46%; 6+ days - 17.02%. Days in the past month where participants' mental and physical health was keeping them from their normal activities were: 0 days - 47.07%; 1-2 days - 31.97%; 3-5 days - 12.43%; 6+ days - 8.53%. Days in the past month where the participants' felt worried, tense, or anxious were: 0 days - 8.02%; 1-2 days - 26.56%; 3-5 days - 23.53%; 6+ days - 41.89%. Days in the past month where the participants' did not get enough sleep were: 0 days - 2.85%; 1-2 days - 10.52%; 3-5 days - 20.68%; 6+ days - 65.95%. Finally, days in the past month where the participants' felt very healthy and full of energy were: 0 days - 1.96%; 1-2 days - 7.86%; 3-5 days - 13.39%; 6+ days - 79.78%.

#### Life Satisfaction

Means and standard deviations were calculated for the BMSLSS-C domains (Table 2). There was little variation among the mean scores for the Friendships, School, Self, Living Environment, and Physical Appearance domains with most falling within "mostly satisfied." The exception was the Family domain, which participants reported being "pleased." Some greater variation was also observed in the Romantic Relationships domain.

**Table 2:** Mean BMSLSS-C scores

	M	*SD*
Family	6.08	1.04
Friendships	5.87	1.05
School	5.29	1.22
Self	5.49	1.19
Environment	5.23	1.32
Romantic Relationships	4.95	1.66
Physical Appearance	4.95	1.24

#### Developmental Assets

Similar to previous research [36,37], a cut-off system was derived to separate the students into those who had specific developmental assets and those who did not. For Family Communication, Peer Role Models, Future Aspirations, Responsible Choices, and Community Involvement, individuals with a score higher than 2 (thereby selecting 2: usually/very important/mostly like you or 3: almost always/extremely important/very much like you) were interpreted as having the asset. With respect to Non-parental Adult Role Models and Cultural Respect/Life, individuals with a score 1 or lower (indicating a selection of 0: strongly agree or 1: agree) would have the asset. In terms of Spirituality, a score of 2 or lower (2: agree and 1: strongly agree) would represent the presence of the asset. Table 3 presents the percent of students with each developmental asset. Over 7% of the students reported possessing none of the developmental assets while 3.9% reported possessing all eight assets.

**Table 3:** Distribution of Developmental Assets

Asset	Mean	SD	% with Asset
1. Family Communication	2.20	.71	73.7
2. Peer Role Models	2.38	.54	86.1
3. Future Aspirations	2.39	.61	84.3
4. Responsible Choices	2.59	.41	95.5
5. Non-parental Adult Role Models	1.23	.67	50.5
6. Spirituality	1.69	1.13	35.9
7. Community Involvement	1.46	.79	30.7
8. Cultural Respect/Life	1.22	.59	51.0

### Structural Equation Models

The final models resulting from the SEM procedures eliminated some items from the developmental asset measure and the HRQOL-14 due to model global fit indices, microfit indices, or parsimony. Items removed from the developmental asset measure were from the constructs of Spirituality ("I am very spiritual" and "I am very religious") and Cultural Respect ("I respect the beliefs of people even if they are of a different race"). Items eliminated from the HRQOL-14 were questions about physical health status and included self-rated health, days of poor physical health, and days full of energy. No items from the BMSLSS-C were eliminated from the final models.

**Figure 1:**
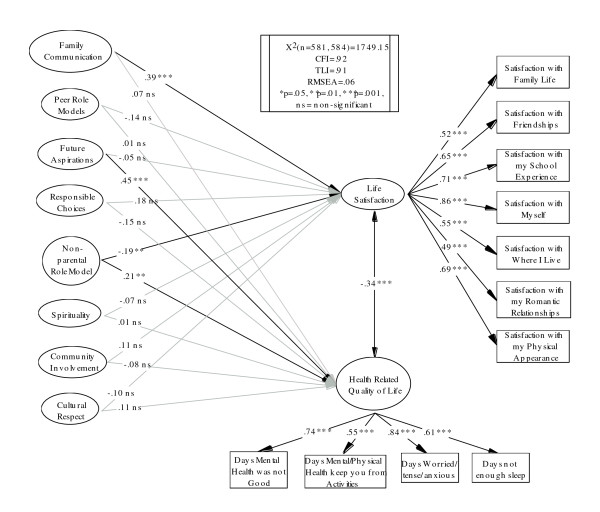
**Final Model of the Developmental Assets' Relationship with Life Satisfaction and HRQOL**.

Variations of the final model as predicted by theory were examined (contact the primary author for a table of results of all models tested). The primary goal of the proposed models was to use the Developmental Assets to simultaneously predict HRQOL and Life Satisfaction. Figure 1 presents the final model and parameter estimates. The final model fit the data well, χ^2^(*n *= 581, 515) = 1252.23, CFI = .94, TLI = .93, RMSEA = .05.

Three significant pathways are demonstrated in Figure 1. First, respondents who reported higher levels of Family Communication also reported higher life satisfaction. Second, individuals who reported higher levels of Future Aspirations also indicated poorer mental HRQOL days. Finally, the third significant pathway in the model suggests that in the sample, the developmental asset of Non-Parental Role Models is indirectly related to decreased life satisfaction and directly related to poor mental HRQOL days.

### Invariance Tests of the Model across Gender

**Figure 2:**
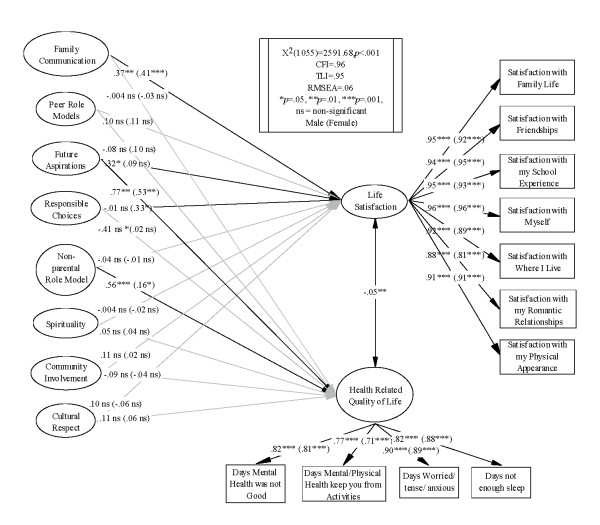
**Model Invariance across Gender**.

Initial tests examined the dependent variables across gender (see Figure 2). In Figure 2, estimates for males and females are provided for each variable, with female estimates in parentheses. Male participants were more likely to report zero poor mental health days than female participants, χ^2^(*n *= 562, 6) = 20.28, *p *= .002. Men and women did not differ significantly on their health keeping them from their daily activities, χ^2^(*n *= 563, 6) = 11.86, *p *= .07. Women reported more days that they felt tense or anxious in comparison to the men, χ^2^(*n *= 561, 6) = 38.07, *p *< .001. There were no gender differences on the number of days not getting enough sleep, χ^2^(*n *= 561, 6) = 2.72, *p *= .84 or on any of the BMSLSS-C items, (Males: *n *= 160, *M *= 38.39, *SD *= 6.42; Females: *n *= 400, *M *= 37.68, *SD *= 5.67), *t*(558) = 1.29, *p *= .20, Cohen's d = .12.

Tests of factorial invariance were performed on the final model, and the overall chi-square was significant, χ^2^(1055) = 2591.68, *p *< .001, CFI = .96, TLI = .95, RMSEA = .06. For both models, the male and female participants' Family Communication predicted Life Satisfaction, and both Future Aspirations and Non-Parental Role Models predicted poor Mental HRQOL. In the male participant model only, Future Aspirations predicted Life Satisfaction. In the female participant model only, Responsible Choices predicted Life Satisfaction. The model examining invariance across male and female participants is presented in Figure 2.

## Discussion

Previous research has explored the relationships between developmental assets and a variety of risk behaviors [7,9-12,36,37,53,54]. This body of research collectively suggests that the greater number of assets an adolescent possesses, the more they are protected against maladaptive behavior. Life satisfaction research [21-23,25,28] also suggests a similar relationship, which is why life satisfaction has been suggested to be an important health outcome [29]. HRQOL is an important protective health construct against behaviors such as unsafe alcohol use in college students [47]. However, no prior research has attempted to examine the concurrent relationships between developmental assets and both life satisfaction and HRQOL. Therefore, understanding how each of these important areas relates to one another has important implications for health promotion practice.

The present study extends the previous literature to college students and offers additional support for the connection between positive family communication and increased life satisfaction. Specifically, positive family support, parental acceptance, and communication are among the most influential factors determining life satisfaction among adolescents [55,56]. In addition, parental support and positive reinforcement help to smooth the transition into college and reduce student levels of loneliness, increase psychological adjustment, and increase academic achievement. Not surprisingly, there is also positive relationship between increased levels of life satisfaction and positive psychosocial functioning [55].

Conversely, the findings pertaining to Non-Parental Role Models and Future Aspirations were somewhat unexpected. The results from this investigation indicate that Non-Parental Role Models had a significant relationship with decreased life satisfaction, as well as a significant positive relationship with poor mental HRQOL days (e.g., days worried or tense). Prior research has indicated that the presence of developmental assets such as Non-Parental Role Models and adult support play an important role in overall adolescent development [57]. Constructive-natured relationships with adults have also been related to positive outcomes in younger samples of adolescents [58,59] and positive outcomes in teenagers.

However, Beam et al. [58] found that high school students who utilized non-parental role models (or "very important people") did not seek these relationships as a means to deal with personal challenges (such as poor mental HRQOL). Rather, it was determined that students formed the associations out of normal daily circumstances. In other words, Beam et al. [58] found that utilization of non-parental adult relationships had very little to do with the student's own personal problems. Although somewhat speculative, the high school environment, where students find themselves in the presence of non-parental adults for the majority of the day, may be different from the college environment where students lead more independent schedules and are older in age. Thus, it is not surprising that school climate research has identified student-adult relationships as one of the most important domains for high school and middle school students [60,61] because students have interactions with non-parental adults as an intrinsic characteristic of their surroundings.

In contrast, students in college settings without specialized arrangements such as faculty-in-residence programs [62] may not have the opportunity to take advantage of such relationships with potential non-parental role models. Programs such as faculty-in-residence programs may offer students a mode of existence that is reminiscent of the naturally occurring environment within high schools. A supplementary point of interest lies in the limitations of student benefits for those relying solely on a formal classroom setting as a medium for interactions. For example, the degree of formality in a collegiate classroom has shown to be less effective [in regards to student outcomes] in comparison to informal interplay between professors and students [63]. However, even with the benefits of casual professor-student relationships documented, methods for novel ways to efficiently encourage these interactions remain a challenge [62]. In sum, interactions with potential non-parental role models in college settings may be naturally less supportive, which may increase poor mental HRQOL days as a result.

The finding in relation to Future Aspirations predicting poor mental HRQOL was also unexpected. Previous research indicates that the development of future aspirations within an individual is correlated with a healthier level of development and lower likelihood of engaging in risk-taking behavior in high school students [10,11]. Quaglia and Cobb [64] define future aspirations as "a student's ability to identify and set goals for the future, while being inspired in the present to work toward those goals." During an adolescent's high school years, serious consideration in regards to future education and occupation often commences [65]. Numerous factors such as media influence (i.e. implied career prestige), family attitudes, academic engagement and achievement, and peer-related gender stereotypes (such as the schema that women become nurses and men become physicians) play a role in the formation of occupational future aspirations [65,66]. Although tentative, college students may harbor a greater temporal urgency for the rigors of the work world and may be more cognizant of the barriers of entry into an increasingly competitive workforce. The educational mission of college may further intensify these concerns in relation to those of high school, which may in turn cause greater worry, anxiety, and loss of sleep, which then reduces mental HRQOL.

## Study Limitations

Limitations to the present study include a Caucasian sample of university students representing one Midwestern university. Additional investigations should be conducted in more diverse university populations in different geographic regions in both urban and rural settings, as findings reported here may yield different results in different populations of university students. In addition, several developmental asset measures contained only two items either to begin with (e.g., Future Aspirations) or because of our model parameters led to item exclusion within a construct (e.g., Cultural Respect/Life). Considering that one of the significant pathways in the final model was Future Aspirations, results here should be interpreted cautiously. Finally, a 56% response rate was achieved in this study, which may limit the generalizability of the findings.

## Conclusions

Guthman et al. [67] found during a 12-year comparative study that college students are experiencing higher levels of mental distress than what was witnessed a decade ago. However, the determination of exactly what catalyzed the increase in rates of severe depression, anxiety and emotional turmoil in college students requires further investigation. The analysis of these findings by colleges and universities may prove useful for the amelioration of current standards which may have encouraged deficiencies in these important facets wellbeing (e.g., school programs which indirectly lead to limited family communication). In addition, developing a deeper understanding of the source of the aforementioned findings is useful in assisting universities with the aim of lowering attrition rates. Decreasing attrition among the student body is a commonly found when student concerns bear a direct influence on the adaptation of campus policies - such as determining standards regarding the quality of dormitory living facilities [68]. It should also be noted here that life satisfaction levels among college students were able to significantly predict academic retention by themselves, but also in combination with overall grade point averages (GPA) 1-3 years in advance [69]. Hence, future studies might seek to extend the models in this study by including longitudinal constructs and retention rates.

Likewise, the results of this study may be utilized for examining the *type *of support non-parental role models are providing students in colleges. While students may have frequent contact with academic advisors and instructional faculty who provide them informational support, students may also need other forms of social support such as emotional (e.g., trust, caring, etc.), instrumental support (aid that directly assists a person in need), and appraisal support (constructive feedback, affirmation, etc.) that have been shown to improve health outcomes [70,71]. Establishing a framework for meetings where multiple forms of support are displayed by non-parental role models at set intervals may not only reestablish the nature of the relationships of those observed in high school students (e.g., [58]), but also may prove useful over potentially waiting for personal problems to develop. Furthermore, the results of this research may help guide effective additional activities, such as community and family support programs [72] which have been established as a fruitful endeavor towards the main goal of improving the overall QOL of the campus student body [73].

## Abbreviations

BMSLSS-C: Brief Multidimensional Students' Life Satisfaction Scale; BRFSS: Behavioral Risk Factor Surveillance System; HRQOL: health-related quality of life; PYD: Positive youth development; QOL: quality of life; SEM: structural equation models; SF-36: Rand Corporation's Short Form-36; US CDC: United States Centers for Disease Control and Prevention

## Competing interests

The authors declare that they have no competing interests.

## Authors' contributions

KJZ conceived and designed the study, collected the data, participated in the analysis and interpretation of the data, and coordinated all aspects of the manuscript. DAT participated in drafting the manuscript. RMW participated in the analysis and interpretation of the data and in drafting the manuscript. All parties have received the manuscript and have reviewed it.
